# Uniportal Thoracoscopic Single-Direction Basal Subsegmentectomy (Left S10a+ci): Trans-Inferior-Pulmonary-Ligament Approach

**DOI:** 10.1245/s10434-021-10806-4

**Published:** 2021-11-11

**Authors:** Chengwu Liu, Wenping Wang, Jiandong Mei, Yunke Zhu, Qiang Pu, Lunxu Liu

**Affiliations:** 1 Department of Thoracic Surgery, West China Hospital, Chengdu, China; 2 Western China Collaborative Innovation Center for Early Diagnosis and Multidisciplinary Therapy of Lung Cancer, Sichuan University, Chengdu, China

**Keywords:** Thoracoscopic, Uniportal, Subsegmentectomy, Lung cancer

## Abstract

Thoracoscopic segmentectomy and subsegmentectomy have been widely accepted for the treatment of peripheral small lung cancers. Thoracoscopic basal subsegmentectomy, especially when performed through a uniportal procedure, is extremely technically challenging, and therefore there are seldom reports of its technical details. In this article, we present a uniportal thoracoscopic left S10a+ci subsegmentectomy following the single-direction strategy through the inferior pulmonary ligament approach.

**Supplementary Information:**

The online version contains supplementary material available at 10.1245/s10434-021-10806-4.

## Background

Thoracoscopic segmentectomy and subsegmentectomy have been widely accepted for the treatment of peripheral small lung cancers, as they have the advantage of preserving postoperative pulmonary function.^1,2^ Thoracoscopic anatomical resections of basal segments are commonly considered much more technically demanding than resections of other segments.^3–6^ Thoracoscopic basal subsegmentectomy is much more complex because of the deep intraparenchymal localization of the subsegmental hilar structures, frequent variations, and complex neighboring relationships of the intersubsegmental planes. Thoracoscopic basal subsegmentectomy performed through a uniportal procedure is one of the most challenging procedures, and therefore there are seldom reports on uniportal thoracoscopic basal subsegmentectomy and its technical details.^7^ We previously described a series of techniques in dealing with thoracoscopic anatomic single or combined basal segmentectomies, including the strategy of single-direction, the trans-inferior-pulmonary-ligament approach, and the stem-branch method for identifying segmental structures.^3, 8–10^ In this context, we would like to share the surgical details of a complex uniportal thoracoscopic basal subsegmentectomy under these surgical strategies and techniques.

## Methods

In this multimedia article, we describe a uniportal thoracoscopic left S10a+ci subsegmentectomy through the inferior pulmonary ligament approach following a single-direction strategy and using the stem-branch method for subsegmental structure tracking (video). High-resolution computed tomography (HRCT) was used to identify the location of the lesion and positional relationship of the subsegmental vessels and bronchi. A 4.5 cm incision was made at the fifth intercostal space across the mid-axillary line. The operation was initiated from the inferior pulmonary ligament. The stem and branches of the basal segmental vein were dissected, and the target branches (intrasubsegmental veins) were tracked and identified based on the branching characteristics recognized on the preoperative HRCT. After transection of the target veins, the target bronchus was dissected and identified, predominantly according to its positional relation to the intersubsegmental vein. The technique of the intracavitary overhanging approach was used to facilitate the placement of an endostapler when dealing with the target bronchus due to the limited operation angle.^11^ The accompanying artery was then identified and divided. Afterwards, the intersubsegmental demarcation line was identified using the inflation–deflation method and the intersubsegmental planes were treated using stapler-based tailoring. Finally, the uniportal thoracoscopic single-direction LS10a+ci subsegmentectomy was completed. Intraoperative frozen pathological examination revealed an adenocarcinoma. We then performed lobe-specific lymph node sampling, with a total of five lymph nodes removed from stations 7, 9, 10, and 13.

## Result

The operation took 125 min, with about 20 mL of blood loss. A chest x-ray on postoperative day 1 revealed that the residual lung re-expanded well. The chest tube was removed on postoperative day 3 and the patient was discharged on postoperative day 4 without any complications. The final pathological examination documented a minimally invasive adenocarcinoma, pT1a(mi)N0M0.

## Discussion

For ground glass opacity (GGO)-dominant subcentimeter lung cancers, no significant differences in oncologic efficacy were observed between wedge resection and anatomic segmentectomy, and the two procedures were mainly selected based on the site of the lesion.^12^ It is generally accepted that lesions at the superficial pulmonary parenchyma can be removed by wedge resection, while those present in deeper locations can be removed by segmentectomy. In this case, the lesion was located in the left posterior basal segment (S10). Although the lesion was superficially located, it was too close to the subsubsegmental bronchi and sufficient margin was hard to achieve if a wedge resection was performed. Theoretically, subsegmentectomy may help preserve more postoperative lung function than segmentectomy because less pulmonary parenchyma is removed. Therefore, we chose to perform a subsegmentectomy for this patient.

We have previously described in detail the technical procedures of single-direction thoracoscopic basal segmentectomies using the trans-inferior-ligament approach and the stem-branch method.^3,8–10^ These techniques enable complex thoracoscopic basal segmentectomies to be performed in a simple manner^3^; however, it is difficult to perform fine anatomy and get a whole view of all the basal subsegmental structures through a uniportal approach. Therefore, when applying these techniques to uniportal procedures, especially to a subsegmentectomy, different technical details are noteworthy. First, the target intrasubsegmental veins are identified, mainly based on the characteristics of the venous branches recognized from the preoperative HRCT. Second, the target subsegmental bronchus is identified based predominantly on the positional relationship between it and the intersubsegmental vein. Third, owing to the limited operation angle, getting an appropriate angle for a stapler via the uniportal approach is challenging. We believe that having an additional port may be helpful for easier stapler passage; however, the technique of the intracavitary overhanging approach that we proposed during uniportal thoracoscopic lobectomy^11^ is helpful enough for us to obtain an appropriate angle for stapler passage when dealing with the bronchus. Therefore, we do not routinely add ports. Finally, an energy device is commonly used for division of the intersubsegmental plane between S6 and S10a because it is difficult to place the stapler appropriately using the uniportal approach.

## Conclusion

Uniportal thoracoscopic basal subsegmentectomy, such as the LS10a+ci illustrated in this paper, can be performed successfully in a simple manner through the trans-inferior-pulmonary-ligament approach following a single-direction strategy. However, it is of great importance that both surgical and oncological issues should be taken into consideration before choosing a subsegmentectomy for lung cancer.

## Supplementary Information

Below is the link to the electronic supplementary material.

Supplementary file 1 (MP4 102863 KB)
